# The Downregulation of MMP23B Facilitates the Suppression of Vitality and Induction of Apoptosis in Endometrial Cancer Cells

**DOI:** 10.1007/s43032-024-01581-0

**Published:** 2024-05-23

**Authors:** Ning Li, Hua Li, Lijuan Wei, Hui Chen, Zhaorong Wu, Si Yuwen, Sufang Yang

**Affiliations:** 1 Department of Gynaecology and Obstetrics, Guangxi International Zhuang Medicine Hospital Affiliated to Guangxi University of Chinese Medicine, Nanning, Guangxi China; 2 Department of Pathology, Guangxi International Zhuang Medicine Hospital Affiliated to Guangxi University of Chinese Medicine, Nanning, Guangxi China; 3 Institute of Basic Medical Science, Medicine and Health Research Institute of Guangxi Academy of Sciences, Nanning, Guangxi China; 4 Graduate School, Guangxi University of Chinese Medicine, Nanning, Guangxi China; 5 Department of Reproductive Health and Infertility, Guangxi International Zhuang Medicine Hospital, Affiliated to Guangxi University of Chinese Medicine, Nanning, Guangxi China

**Keywords:** Endometrial cancer, MMP23B, Survival prognosis, Apoptosis factor

## Abstract

Endometrial cancer is a malignant tumor that commonly occurs in the female reproductive system and its incidence is still increasing. The mechanism of the development of endometrial cancer has not yet been fully clarified, so we need to continuously study the relevant mechanisms of endometrial cancer and continue to explore its biomarkers in order to discover more precise and effective treatment methods for endometrial cancer. RT-qPCR (Real-Time quantitative Polymerase Chain Reaction) experiments were used to detect the expression level of MMP23B (Matrix Metalloproteinase 23B) in endometrial cancer cells; the clinical data of the TCGA (The Cancer Genome Atlas) database were downloaded, and gene expression profiles were analyzed to investigate the correlation between MMP23B (Matrix Metalloproteinase 23B) and the survival prognosis of endometrial cancer, and functional enrichment analysis was performed on MMP23B (Matrix Metalloproteinase 23B) related genes. After silencing MMP23B (Matrix Metalloproteinase 23B), CCK8 (Cell Counting Kit-8), RT-qPCR (Real-Time quantitative Polymerase Chain Reaction), scratch assay, and transwell assay were used to detect cell viability, levels of apoptotic factors, migration rate, and invasion number of endometrial cancer, respectively. MMP23B (Matrix Metalloproteinase 23B) was highly expressed in endometrial cancer, which is closely related to a poor survival prognosis for endometrial cancer, and may act on endometrial cancer through apoptosis-related functions. The downregulation of MMP23B (Matrix Metalloproteinase 23B) reduced the cell viability of endometrial cancer cells, upregulated the expression levels of CASP3 (Caspase-3), CASP8 (Caspase-8) and CASP9 (Caspase-9) in cells, and inhibited cell migration and invasion.

## Introduction

Endometrial cancer is a malignant tumor that commonly occurs in the female reproductive system with significant heterogeneity [1]. Endometrial cancer ranks second in incidence among female reproductive system tumors and third in mortality worldwide [2]. In 2020, there were 417,000 new cases and 97,000 deaths of endometrial cancer [3]. Among them, North America, Europe, Micronesia/Polynesia and Australia/New Zealand had the highest incidence rate, while most of Africa and Central and South Asia had the lowest incidence rate. And it is expected that the incidence of endometrial cancer will significantly increase by 2025 [4, 5]. Current treatment options mainly include surgery, chemotherapy, and radiotherapy [6]. Although the survival rate of early-stage endometrial cancer patients is still relatively optimistic, due to limited treatment options and the high metastatic and recurrent nature of endometrial cancer, the overall survival rate of advanced-stage endometrial cancer patients is low, and the prognosis is not good [7]. The application of hysterectomy combined with radiotherapy and lymph node dissection significantly reduces the mortality of endometrial cancer, but the prognosis and survival rate of advanced and metastatic endometrial cancer patients remain poor [8]. Therefore, clarifying the molecular mechanisms of endometrial cancer occurrence and progression, developing effective treatment strategies, and improving the survival rate of endometrial cancer patients to improve the prognosis are of great significance. In recent years, research on epigenetics has opened up new avenues for exploring the pathogenesis and treatment of endometrial cancer.

MMPs (Matrix Metalloproteinases) are a widely distributed group of membrane proteins with a high degree of structural homology [9]. They are classified as secretory or membrane-bound zinc endopeptidases and are named due to their containing zinc and calcium ions. They are highly conserved in natural evolution and widely distributed in organisms, where they degrade extracellular matrix and facilitate cell migration to other sites [10]. MMPs (Matrix Metalloproteinases) play a crucial role in many cell biological behaviors. MMPs (Matrix Metalloproteinases) expression is low in normal tissue but increases in important physiological and pathological processes of angiogenesis and disease development (such as arthritis, tumor growth and metastasis) [11]. With the deepening research on the MMPs (Matrix Metalloproteinases) system, their role in various malignant tumors has become a hot topic [12]. For example, MMP2 (Matrix Metalloproteinase 2) participates in ECM (Extracellular Matrix) degradation and plays an important role in physiological and pathological processes [13]. It plays a crucial role in tumor invasion and metastasis [14]. MMP9’s (Matrix Metalloproteinase 9) key involvement in ECM (Extracellular Matrix) remodeling highlights its significant correlation with the pathogenesis and progression of cancer at every stage [15]. MMP7 (Matrix Metalloproteinase 7) expression is a validated independent prognostic factor in urothelial carcinoma [16]. MMP22 (Matrix Metalloproteinase 22) and MMP29 (Matrix Metalloproteinase 29) are independent risk factors for human coronary heart disease. MMP2 (Matrix Metalloproteinase 2) serum protein levels are elevated in endometrial cancer patients [17]. Regulating MMP2/1 (Matrix Metalloproteinase 2/1) can promote the proliferation and invasion of endometrial cancer cells [17, 18]. The expression and protein level of MMP23B (Matrix Metalloproteinase 23B) in blood and urine are associated with bladder cancer [19]. Upregulation of MMP23B (Matrix Metalloproteinase 23B) is significantly associated with higher tumor stages in colorectal cancer [20]. However, there is no report yet about the role and mechanism of MMP23B (Matrix Metalloproteinase 23B) in endometrial cancer. Therefore, we used cell biology and bioinformatics analysis to explore the role and possible mechanism of MMP23B (Matrix Metalloproteinase 23B) in endometrial cancer.

## Method

### Cell Culture and Transfection

Human endometrial cancer cells (Ishikawa cells) and endometrial epithelial cells were purchased from iCell Bioscience Inc. (SaiBaiKang (Shanghai) Biotechnology Co., Ltd.). They were cultured in a special cell culture medium (iCell Bioscience Inc.). The cells in logarithmic growth phase were digested with trypsin, collected and counted. The cells were inoculated into 6-well plates, and when the cell growth confluence reached 50%, transfection was carried out. The specific operation steps were referred to the Lipofectamine 3000 transfection reagent instructions. The cells transfected with MMP23B-siRNA (Matrix Metalloproteinase 23B-Small Interfering RNA) were named as si-MMP23B (small interfering RNA targeting Matrix Metalloproteinase 23B) group, the cells transfected with RNA (Ribonucleic Acid) Control were named as NC (Negative Control) group, and the blank control group was the cells without transfection.

### RT-qPCR

The total RNA (Ribonucleic Acid) was extracted from the cells using TriQuick Reagent (R1100, Solarbio, China). Reverse transcription reaction was carried out using MonScript™ RTIII All-in-One Mix with dsDNase (Monad, China) according to the instructions. qPCR reaction was performed in 2x S6 Universal SYBR qPCR mix-V3 reagent kit (NovaBio, China). Specific PCR (Polymerase Chain Reaction) primers of MMP23B (Matrix Metalloproteinase 23B), CASP3 (Caspase-3), CASP8 (Caspase-8) and CASP9 (Caspase-9) were purchased from Sangon Biotech (Shanghai) Co., Ltd. MMP23B (Matrix Metalloproteinase 23B): Forward primer TGGTACAAGGACCAGGAGCC, Reverse primer TTGGCGATGATGCTCAGGTG. CASP3 (Caspase-3): Forward primer CATACTCCACAGCACCTGGTTA, Reverse primer ATGG CACAAAGCGACTGGAT. CASP8 (Caspase-8): Forward primer AGAAACTTCTTCCTGGGAGCC, Reverse primer CAGGAGAATATAATCCGCTCCA. CASP9 (Caspase-9): Forward primer CACTGGCTCCAACATCGACT, Reverse primer AGCCAGCACCATTTTCTTGG. GAPDH (Glyceraldehyde 3-Phosphate Dehydrogenase): Forward primer GACAGTCAGCCGCATCTTCT, Reverse primer G CGCCCA ATACGACCAAATC.

### Survival Analysis

Downloaded the clinical data and gene expression data of endometrial cancer in the TCGA database (The Cancer Genome Atlas database), and used the R language survival package and survminer package to draw KM (Kaplan-Meier) curves to analyze the correlation between MMP23B (Matrix Metalloproteinase 23B) and endometrial cancer survival in the past. Also, a multi-factor cox regression analysis was conducted to identify independent factors that affected endometrial cancer survival in the past, and the forestplot package was used to draw a forest plot.

### Functional Enrichment Analysis

Based on the gene expression data of endometrial cancer in the TCGA database (The Cancer Genome Atlas database), the R language limma package was used to analyze the associated genes of MMP23B (Matrix Metalloproteinase 23B) (according to the gene expression level of MMP23B, it was divided into a low-expression group and a high-expression group, and the differentially expressed genes in the two groups were analyzed). The R language clusterProfiler, org.Hs.eg.db packages, and other tools were used to perform functional enrichment analysis on the associated genes.

### CCK8

Logarithmically phase Ishikawa cells were dissociated and resuspended, and then seeded at a density of 4 × 10^3^ cells per well into a 96-well plate. The plate was placed in a CO_2_ (Carbon Dioxide) incubator for culture. After 24 h, the cells were fully adhered, and the medium was discarded. Fresh medium was added for transfection, and then incubated. After 48 h, CCK-8 (Cell Counting Kit-8) reagent was added to each well, and the plate was placed in the incubator for 1–4 h. The absorbance at 450 nm was measured using an enzyme-linked immunosorbent assay (ELISA) reader.

### Scratch Assay

Taking Ishikawa cells in the logarithmic growth phase, they were fully digested to obtain a uniform cell suspension. The suspension was counted, and cells were inoculated into a 6-well plate at a density of 5 × 10^5^ cells/well. The plate was marked with horizontal lines on the outside of the bottom of each well. Cells were transfected when the confluence reached 80-90%. After 24 h of transfection, the cell density was 95-100%. Vertical lines were drawn in each well of the 6-well plate using a 200 µL pipette tip perpendicular to the horizontal lines, and PBS was used to wash until no cells were shed at the scratched area. Cells were then cultured in serum-free medium. Photographs were taken at 0 h, 24 h, and 48 h after scratching.

### Transwell Assay

The cells were inoculated in front of the Transwell chamber. The matrix gel was diluted with serum-free medium in a specific ratio and added to the upper chamber of the Transwell apparatus, creating a gel model of extracellular matrix on the polycarbonate membrane. The intervened cells were then inoculated into the Transwell chamber coated with matrix gel using serum-free medium, while complete culture medium was slowly added to the lower chamber. At the specified time point, the culture plate was removed, and the cells and extracellular matrix gel on the upper surface of the basement membrane that did not penetrate the membrane were gently wiped off with a cotton swab. After washing with PBS (Phosphate Buffered Saline), the cells that had penetrated the membrane in each chamber were fixed and stained, and the number of cells that had penetrated the membrane in each chamber was observed under a microscope.

### Statistical Analysis

GraphPad Prism 8 was used for statistical analysis and visualization of experimental data. Experimental data were expressed as mean ± standard deviation. t test with Welch’s correction was used for comparison between two groups, and analysis of variance was used for comparison between multiple groups. *P* < 0.05 was considered statistically significant.

### Result

### MMP23B Overexpression in Endometrial Cancer

**Fig. 1:**
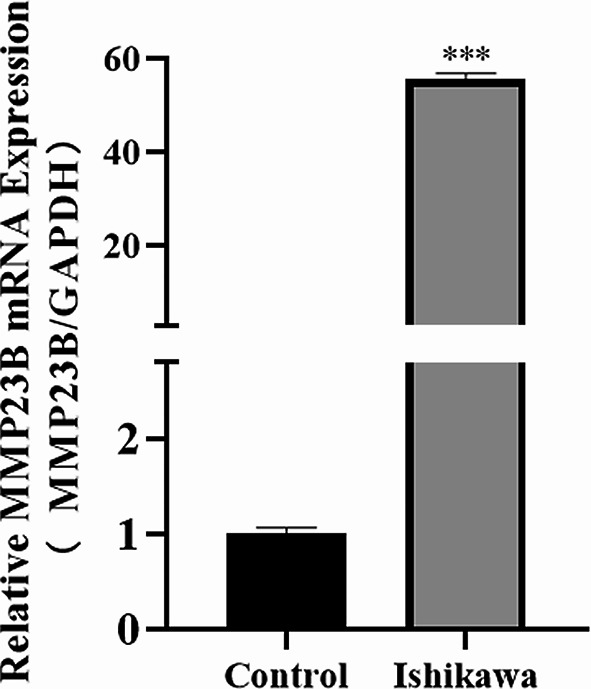
Expression levels of MMP23B in Ishikawa cells and endometrial epithelial cells (Control) (*Note: Compared to the Control group, ***P < 0.001)

The expression levels of MMP23B (Matrix Metalloproteinase 23B) in human endometrial cancer Ishikawa cells and endometrial epithelial cells were detected using RT-qPCR (Reverse Transcription Quantitative Polymerase Chain Reaction). The results showed that the expression of MMP23B (Matrix Metalloproteinase 23B) was higher in endometrial cancer than in endometrial epithelial cells (*P* < 0.05) (Fig. 1).

### MMP23B Closely Associated with Survival Prognosis in Endometrial Cancer

**Fig. 2:**
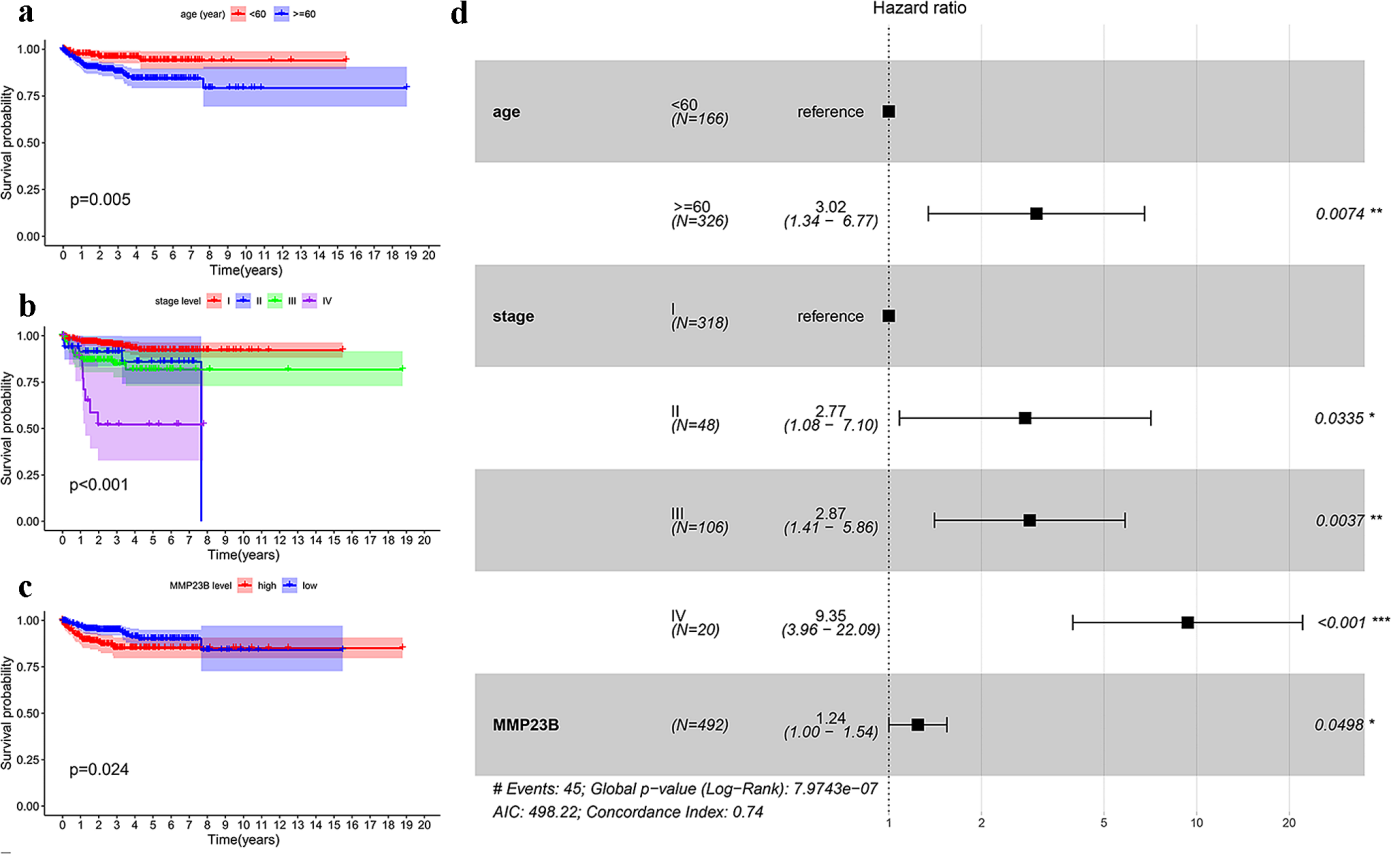
Factors influencing survival prognosis in endometrial cancer. (**a**-**c**): Kaplan-Meier (KM) survival curves for age, stage, and MMP23B in endometrial cancer. (The blue curve represents patients with low expression of MMP23B in endometrial cancer, while the red curve represents patients with high expression of MMP23B. (**d**): Multivariate Cox regression forest plot

Kaplan-Meier curves (Fig. 2a-c) demonstrated that age, stage, and MMP23B expression are associated with survival in endometrial cancer (*P* < 0.05). Specifically, at around 7 years of survival time, patients with high MMP23B (Matrix Metalloproteinase 23B) expression had lower survival rates, indicating that high levels of MMP23B (Matrix Metalloproteinase 23B) expression are associated with poor prognosis in endometrial cancer.

To further investigate the factors affecting the survival prognosis of endometrial cancer and to analyze whether MMP23B (Matrix Metalloproteinase 23B) is an independent prognostic factor, we conducted a multivariate Cox regression analysis including age, stage, and MMP23B (Matrix Metalloproteinase 23B). The results showed that age (*P* < 0.05, HR = 3.02), stage (*P* < 0.05, HR = 2.77, 2.87, 9.35), and MMP23B (Matrix Metalloproteinase 23B) (*P* < 0.05, HR = 1.24) are all independent prognostic factors for endometrial cancer (Fig. 2d).

### MMP23B Functional Enrichment Results in Endometrial Cancer

MMP23B (Matrix Metalloproteinase 23B) is associated with biological processes such as negative regulation of extrinsic apoptotic signaling pathway and epidermal growth factor receptor signaling pathway in endometrial cancer, related to molecular functions like proteoglycan binding, linked to cellular components such as collagen-containing extracellular matrix and immunoglobulin complex, and linked to KEGG (Kyoto Encyclopedia of Genes and Genomes) pathway such as Protein digestion and absorption (Table 1).

**Table 1:** MMP23B functional enrichment results in endometrial cancer

ONTOLOGY	ID	Description	pvalue	*p*.adjust
BP	GO:2,001,237	negative regulation of extrinsic apoptotic signaling pathway	0.026	0.042
BP	GO:2,001,240	negative regulation of extrinsic apoptotic signaling pathway in absence of ligand	0.009	0.027
BP	GO:0007173	epidermal growth factor receptor signaling pathway	0.030	0.045
BP	GO:0045229	external encapsulating structure organization	0.003	0.021
BP	GO:0043062	extracellular structure organization	0.003	0.021
MF	GO:0043394	proteoglycan binding	0.010	0.028
CC	GO:0062023	collagen-containing extracellular matrix	0.004	0.017
CC	GO:0019814	immunoglobulin complex	0.027	0.049
CC	GO:0071745	IgA immunoglobulin complex	0.003	0.016
KEGG	hsa04974	Protein digestion and absorption	0.024	0.048
KEGG	hsa04672	Intestinal immune network for IgA production	0.011	0.048
KEGG	hsa04512	ECM-receptor interaction	0.021	0.048

**Fig. 3:**
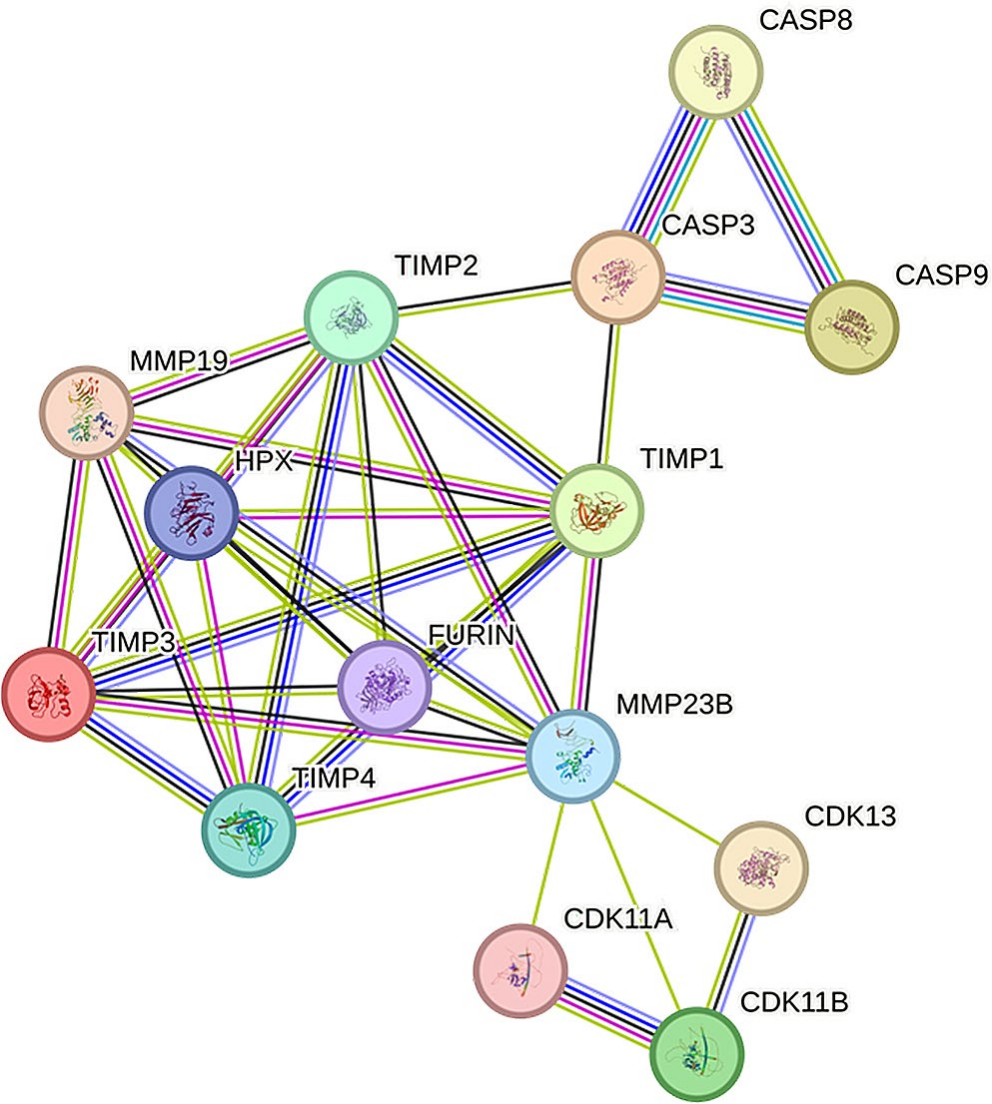
The protein interaction network between MMP23B and apoptosis factors in the STRING database

Although MMP23B (Matrix Metalloproteinase 23B) was found to be potentially associated with apoptosis-related pathways in functional enrichment analysis, we wanted to explore whether there is any correlation between MMP23B (Matrix Metalloproteinase 23B) and apoptosis factors in protein interactions. We input MMP23B (Matrix Metalloproteinase 23B) into the STRING database (Search Tool for the Retrieval of Interacting Genes database) and added CASP3 (Caspase-3), CASP8 (Caspase-8), and CASP9 (Caspase-9) to the MMP23B (Matrix Metalloproteinase 23B) protein interaction network. We discovered that these three apoptosis factors are connected to MMP23B (Matrix Metalloproteinase 23B) through MMPs inhibitors TIMP1 and TIMP2 (Tissue Inhibitor of Metalloproteinase 1 and 2) (Fig. 3).

### Validation of the Silencing Effect of MMP23B

**Fig. 4:**
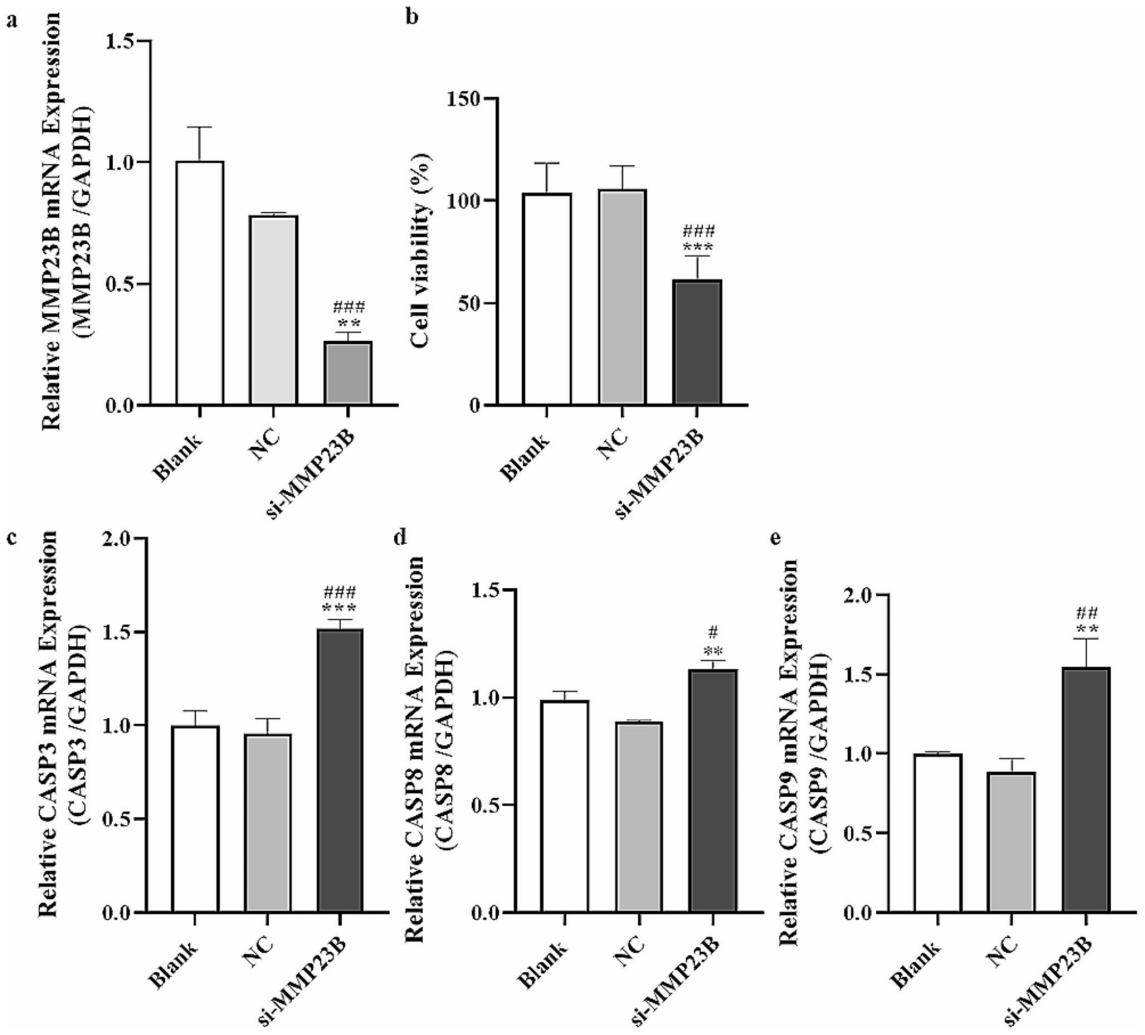
Inhibition of endometrial cancer cells by downregulation of MMP23B. (**a**): Expression of MMP23B in the blank, NC, and si-MMP23B groups. (**b**): Comparison of cell viability in the blank, NC, and si-MMP23B groups. (**c**-**e**): Expression of CASP3, CASP8, and CASP9 in the blank, NC, and si-MMP23B groups. Note: Compared to the Blank group, ^#^*P* < 0.05, ^##^*P* < 0.01, ^###^*P* < 0.001; Compared to the NC group, *P < 0.05, **P < 0.01, ***P < 0.001

Silencing of MMP23B (Matrix Metalloproteinase 23B) in endometrial cancer cells was validated by RT-qPCR (Reverse Transcription Quantitative Polymerase Chain Reaction) detection of MMP23B (Matrix Metalloproteinase 23B) expression in endometrial cancer. Results showed that the expression level of MMP23B (Matrix Metalloproteinase 23B) in the si-MMP23B (small interfering RNA targeting Matrix Metalloproteinase 23B) group was significantly lower than that in the blank and NC (Negative Control) groups (*P* < 0.05) (Fig. 4a).

### Downregulation of MMP23B Inhibits the Viability of Endometrial Cancer Cells

After transfection for 48 h, the CCK-8 (Cell Counting Kit-8) experiment results indicated that the viability of endometrial cancer cells in the si-MMP23B (small interfering RNA targeting Matrix Metalloproteinase 23B) group was significantly lower than that in the blank and NC (Negative Control) groups (*P* < 0.05) (Fig. 4b).

### Downregulation of MMP23B Promotes Upregulation of Apoptotic Factors in Endometrial Cancer Cells

The RT-qPCR (Reverse Transcription Quantitative Polymerase Chain Reaction) results showed that the expression levels of CASP3 (Caspase-3), CASP8(Caspase-8), and CASP9(Caspase-9) in the si-MMP23B (small interfering RNA targeting Matrix Metalloproteinase 23B) group were significantly higher than those in the blank and NC (Negative Control) groups (*P* < 0.05) (Fig. 4c-d).

### Downregulation of MMP23B Inhibits the Migration of Ishikawa Cells

**Fig. 5:**
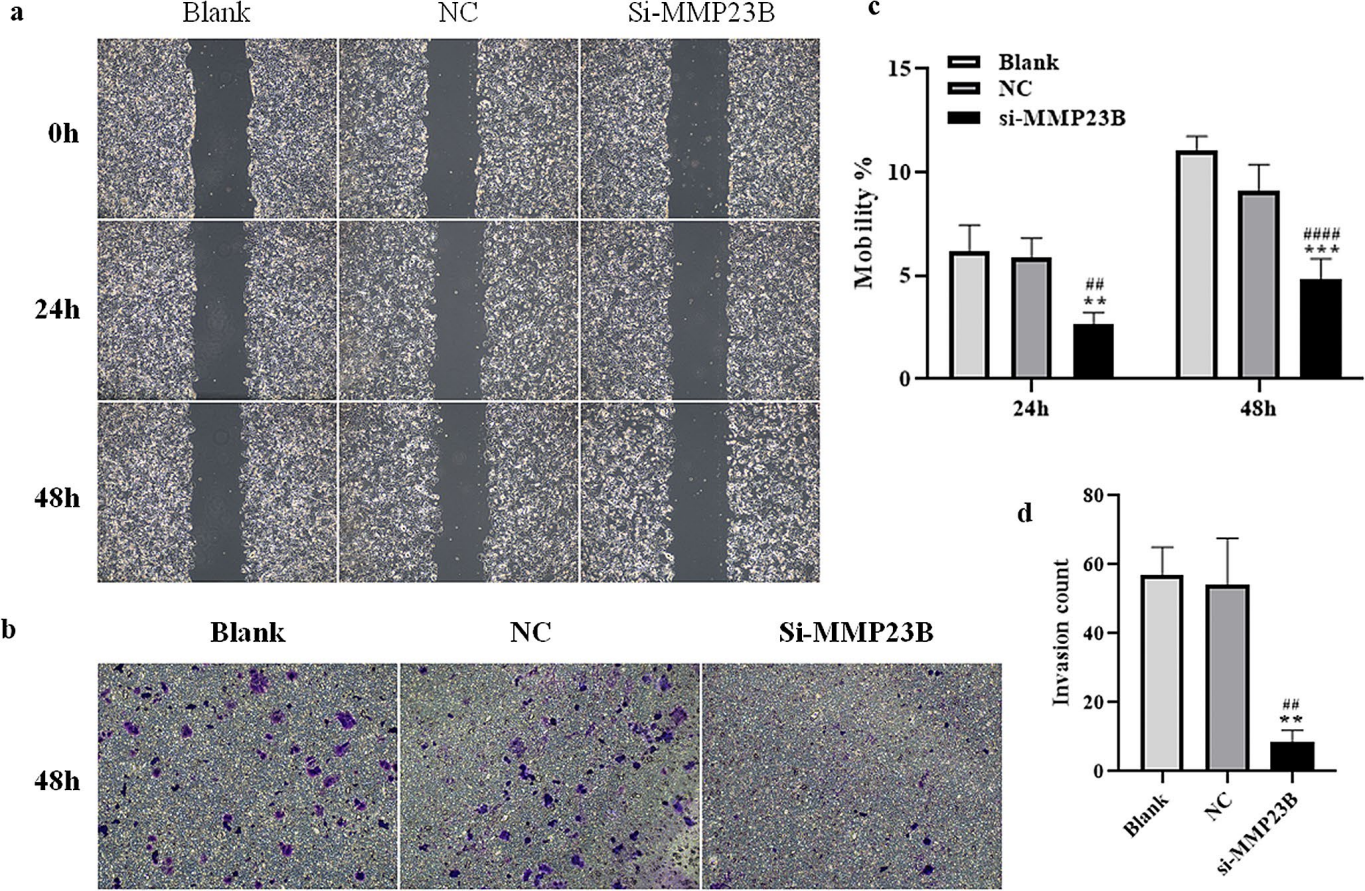
The effect of silencing MMP23B on the migration and invasion of Ishikawa cells. (**a**-**b**): Cell migration and migration rate statistics of the Blank group, si-NC group, and si-MMP23B group at 0 h, 24 h, and 48 h. (**c**-**d**): Cell invasion and invasion number statistics of the Blank group, si-NC group, and si-MMP23B group at 48 h. Note: Compared with the Blank group, #*P* < 0.05, ##*P* < 0.01, ###*P* < 0.001 and ####*P* < 0.0001; compared with the NC group, *P < 0.05, **P < 0.01, ***P < 0.001 and ****P < 0.0001

The scratch assay showed that (Fig. 5a-b), both at 24 h and 48 h, the cell migration rate of the si-MMP23B (small interfering RNA targeting Matrix Metalloproteinase 23B) group was significantly lower than that of the Blank group and the si-NC (siRNA Negative Control) group (*P* < 0.05); while there was no significant difference in the cell migration rate between the Blank group and the si-NC (siRNA Negative Control) group (*P* > 0.05).

### Downregulation of MMP23B Inhibits Invasion of Ishikawa Cells

The transwell assay results showed that the number of invasive cells in the three groups was low at 24 h, so it was not statistically significant. At 48 h (Fig. 5c-d), the number of invasive cells in the si-MMP23B (small interfering RNA targeting Matrix Metalloproteinase 23B) group was significantly lower than that in the Blank group and the si-NC (siRNA Negative Control) group (*P* < 0.05); while there was no significant difference in the number of invasive cells between the Blank group and the si-NC (siRNA Negative Control) group (*P* > 0.05).

## Discussion

Currently, the incidence and mortality rates of endometrial cancer are increasing year by year, and there is a trend towards a younger age of onset. There is an urgent need for further in-depth exploration of the different regulatory mechanisms and relationships of endometrial cancer in order to provide new targets and strategies for clinical treatment, to provide a theoretical basis for drug development from the perspective of molecular mechanisms, and to provide new clues for the clinical treatment of endometrial cancer.

In recent decades, due to the involvement of MMPs (Matrix Metalloproteinases) in many physiological and pathological processes, there has been overwhelming growth in scientific interest in these enzymes. MMPs (Matrix Metalloproteinases) are a group of zinc-dependent endopeptidases with high heterogeneity [21]. After synthesis as inactive zymogens, MMPs (Matrix Metalloproteinases) are activated during the process of extracellular secretion. Activated MMPs (Matrix Metalloproteinases) can degrade almost all components of the ECM (Extracellular Matrix) and regulate multiple signaling pathways by activating or inactivating proteases, cytokines, cell surface receptors, and other substrates [22]. Slight imbalances in MMPs (Matrix Metalloproteinases) levels or activity can lead to pathologies such as vascular disease [23], autoimmune diseases [24], and cancer [25]. In cancer, many MMPs (Matrix Metalloproteinases) are upregulated, regulating ECM (Extracellular Matrix) remodeling, promoting tumor invasion, and ultimately driving tumor progression [26]. For example, MMP13 (Matrix Metalloproteinase 13) secreted by fibroblasts promotes tumor angiogenesis [27], while MMP2 (Matrix Metalloproteinase 2) and MMP9 (Matrix Metalloproteinase 9) degrade ECM (Extracellular Matrix) components to promote metastasis [28]. Transient activity of MMP28 (Matrix Metalloproteinase 28) in A549 lung adenocarcinoma cells also induces TGF-β-dependent EMT (Epithelial-to-Mesenchymal Transition), leading to atypical proliferation and invasive tendencies of cancer cells [29]. Notch3 also induces the formation of metastatic foci in pancreatic ductal adenocarcinoma cells through MMP2 (Matrix Metalloproteinase 2) and MMP9 (Matrix Metalloproteinase 9) [30]. NKILA (NF-κB Interacting Long Noncoding RNA Antisense to NF-κB p65) mediates the phosphorylation of I-κB α and the translocation of NF-κB to the nucleus, thereby reducing the expression of MMP14 (Matrix Metalloproteinase 14), impairing the migration and invasion of ESCC (Esophageal Squamous Cell Carcinoma) cells [31].

MMP23B (Matrix Metalloproteinase 23B) has been less reported to be involved in cancer within this family. The expression level of MMP23B (Matrix Metalloproteinase 23B) in primary glioblastoma patients is significantly higher in glioblastomas than in non-tumorous white matter [32]. MMP23B (Matrix Metalloproteinase 23B) is significantly increased in IL-11-treated JEG-3 cells [33, 34], and silencing MMP23B (Matrix Metalloproteinase 23B) expression leads to a significant decrease in the invasion of JEG-3 cells at baseline and after IL-11 treatment. The expression and protein levels of MMP23B (Matrix Metalloproteinase 23B) in blood and urine are associated with bladder cancer [19]. Genes such as MMP23B (Matrix Metalloproteinase 23B) are related to immune infiltration in pancreatic neuroendocrine tumors [35]. MMP23B (Matrix Metalloproteinase 23B) functions through the tumor necrosis factor (TNF) signaling pathway. Northern blot analysis shows that MMP23 (Matrix Metalloproteinase 23) is mainly expressed in the ovary, testis, and prostate, suggesting a specific role for this MMPs (Matrix Metalloproteinases) in the reproductive process [33]. MMP23B (Matrix Metalloproteinase 23B) can promote the invasiveness of MDA-MB-231 breast cancer cells [36].

We believe that MMP23B (Matrix Metalloproteinase 23B) may be involved in the relevant regulation of the occurrence and development of endometrial cancer. Therefore, we used RT-qPCR to detect the expression level of MMP23B (Matrix Metalloproteinase 23B) in Ishikawa cells. Ishikawa cells are a human endometrial adenocarcinoma cell line commonly used to study the mechanisms, drug sensitivity, and treatment methods of endometrial cancer. The results showed high expression of MMP23B (Matrix Metalloproteinase 23B) in endometrial cancer. At this point, we do not yet understand the function of MMP23B (Matrix Metalloproteinase 23B) in endometrial cancer. Because compared to normal cells, tumor cells produce hundreds of differentially expressed genes that are distinct from normal cells, most of which may not play a critical role in tumorigenesis. At this time, we downloaded important data on gene expression profiles and clinical data of endometrial cancer from the TCGA database (The Cancer Genome Atlas database) and analyzed factors such as MMP23B (Matrix Metalloproteinase 23B) that may be related to the prognosis of endometrial cancer. We found that patients with low expression of MMP23B (Matrix Metalloproteinase 23B) had a higher survival rate around 7 years, and it was an independent risk factor for the survival of endometrial cancer, indicating that high expression of MMP23B (Matrix Metalloproteinase 23B) is associated with poor prognosis of endometrial cancer, and suggesting that MMP23B (Matrix Metalloproteinase 23B) may be a potential biomarker for the treatment of endometrial cancer.

Cancer is a progressive disease that goes through multiple stages, each associated with specific molecular, genetic, and cellular changes, enabling it to acquire increasingly malignant phenotypes. Progression from one stage to the next is driven by a process of spontaneous genetic variation and selection. As subtypes of tumor cells with malignant characteristics gain a competitive advantage, their numbers increase continuously. Ultimately, through multiple mutations, selection, and amplification, tumor cells evolve from early benign tumors into lethal cancers. This process occurs only with sustained cell proliferation and cell survival. Therefore, we decided to verify whether MMP23B (Matrix Metalloproteinase 23B) is involved in and regulates the proliferation and apoptosis signal transduction of endometrial cancer cells. We first conducted a CCK8 (Cell Counting Kit-8) experiment and found that after silencing MMP23B (Matrix Metalloproteinase 23B), its expression level decreased, leading to a decrease in the cell viability of endometrial cancer. The abnormal proliferation capacity of tumor cells is related to the enhancement of cell viability, indicating that low levels of MMP23B (Matrix Metalloproteinase 23B) reduce the proliferation capacity of endometrial cancer cells.

The occurrence and development of cancer involve the excessive proliferation of cancer cells, and to maintain the health of the body, cancer cells must be induced to undergo apoptosis. Apoptosis is a process involving multiple factors and pathways, and cannot be judged based on a single index. The Caspase family plays a functional role in many pathways of apoptosis signal transduction. CASP3 (Caspase-3) is a key executioner molecule in cell apoptosis. When cells receive a signal of apoptosis, CASP3 (Caspase-3) is activated, leading to the cleavage of specific proteins, causing structural damage and cell death. CASP8 (Caspase-8) is an executor of the extrinsic apoptosis pathway. When cells receive death signals from the outside, such as through receptors binding death ligands, CASP8 (Caspase-8) is activated. After activation, CASP8 (Caspase-8) will further activate downstream CASP3 (Caspase-3), inducing cell apoptosis. CASP9 (Caspase-9) is an executor of the intrinsic apoptosis pathway. When factors such as DNA damage, hypoxia, or metabolic stress induce cell apoptosis, CASP9 is activated. After activation, CASP9 (Caspase-9) will also activate downstream CASP3 (Caspase-3), inducing cell apoptosis. In summary, CASP3 (Caspase-3), CASP8 (Caspase-8), and CASP9 (Caspase-9) play crucial roles in the process of cell apoptosis. Our experiments show that after silencing MMP23B (Matrix Metalloproteinase 23B), the apoptosis factors CASP3 (Caspase-3), CASP8 (Caspase-8), and CASP9 (Caspase-9) in endometrial cancer cells significantly increase, suggesting that downregulation of MMP23B (Matrix Metalloproteinase 23B) expression promotes the apoptosis of endometrial cancer cells. Moreover, our experiment also confirmed that downregulation of MMP23B (Matrix Metalloproteinase 23B) can inhibit the migration and invasion of endometrial cancer cells.

Based on the above, we believe that MMP23B (Matrix Metalloproteinase 23B) may be a therapeutic target for endometrial cancer. There have been many studies on the development of MMPs (Matrix Metalloproteinases) inhibitors, which have great potential value in clinical applications. Current research shows that endometrial cancer expresses more TIMPs (Tissue Inhibitors of Metalloproteinases), including MMP26 (Matrix Metalloproteinase 26), TIMP-3 (Tissue Inhibitor of Metalloproteinases-3), and TIMP-4(Tissue Inhibitor of Metalloproteinases-4) [37]. MMP23 (Matrix Metalloproteinase 23) is a potential immunosuppressive target in melanoma, as well as a potential biomarker for providing information on immunotherapy for melanoma [38]. Research also shows that individual genes of MMPs (Matrix Metalloproteinases) or TIMPs (Tissue Inhibitors of Metalloproteinases) have limited accuracy as predictors of HNSCC (Head and Neck Squamous Cell Carcinoma) progression and prognosis, although some studies suggest that MMPs (Matrix Metalloproteinases) are driving factors in cancer progression [39].

Further researches should be conducted on tissue samples from the patients to evaluate atypical endometrial hyperplasia and observe whether the expression of MMP23B has also changed in these tissues. Additionally, it may be valuable to assess the potential relationship between the expression characteristics of MMP23B and the FIGO stage of malignant cases using prognosis data. It has been reported that gradual increments in MMP9 expression may indicate progression from normal to hyperplastic and to low- and high-grade cancerous endometrium [40]. Therefore, investigating whether the expression of MMP23B increases with tumor grading and whether it is also involved in the progression of endometrial cancer is with highly clinical value.

In any case, our research indicates that MMP23B (Matrix Metalloproteinase 23B) is a potential therapeutic target for endometrial cancer, providing new ideas for the use of matrix metalloproteinase inhibitors in the treatment of endometrial cancer.

## Data Availability

Not applicable.
